# Editorial: Novel techniques for colorectal cancer

**DOI:** 10.3389/fsurg.2026.1814864

**Published:** 2026-03-18

**Authors:** Mauro Podda, Baohong Yang

**Affiliations:** 1 Department of Surgical Science, Universita Degli Studi di Cagliari, Cagliari, Italy; 2 Department of Oncology, Capital Medical University Daxing Teaching Hospital, Beijing, China

**Keywords:** colorectal cancer, CRS-HIPEC, liver metastasis, machine learning, multidisciplinary care, peritoneal metastasis, stereotactic body radiotherapy, surgical oncology

## Abstract

Colorectal cancer (CRC) remains a leading cause of cancer-related mortality worldwide. Despite significant advancements in systemic therapies, the management of locally advanced and metastatic disease, including peritoneal and liver metastases, continues to present complex clinical challenges. The evolution of treatment paradigms from purely systemic approaches to aggressive, multimodal strategies underscores the necessity for continuous innovation in surgical techniques, targeted therapies, and diagnostic tools. In this research topic, we present a collection of four insightful articles that address critical aspects of CRC management. These contributions span the spectrum of care, from refining surgical safety and exploring novel combination therapies to leveraging machine learning for early detection, thereby reflecting the multidisciplinary nature of modern oncologic care.

## Surgical innovation and intraoperative safety

Surgical management remains the cornerstone of treatment with curative intent. The high risk of peritoneal dissemination in locally advanced stages has driven the exploration of intraoperative adjunctive therapies to improve long-term outcomes. The randomized controlled trial by Jin et al. evaluated the safety and feasibility of intraoperative intraperitoneal perfusion chemotherapy (IPC) with raltitrexed during laparoscopic radical resection. Their findings suggest that this combined approach is not only feasible but exhibits an acceptable short-term safety profile. Furthermore, the exploratory observation of a significant decrease in carcinoembryonic antigen (CEA) levels in the IPC group highlights the potential of localized chemotherapy to eliminate free cancer cells and microscopic deposits, offering a promising prophylactic strategy to mitigate the risk of peritoneal metastasis in high-risk patients Jin et al.. Although preliminary, the results of this study provide a crucial foundation for larger trials aimed at validating the long-term survival benefits of intraperitoneal chemotherapy in the adjuvant setting.

## Managing advanced peritoneal disease

The challenge of treating established peritoneal metastasis is addressed by Karimi et al. in a comprehensive review. Cytoreductive surgery (CRS) combined with hyperthermic intraperitoneal chemotherapy (HIPEC) has emerged as a pivotal treatment for selected patients. However, this aggressive approach is associated with significant morbidity. The authors systematically categorize the spectrum of complications (surgical, anesthetic, and chemotherapy-related) and analyze the risk factors influencing clinical outcomes. This review emphasizes that while CRS + HIPEC offers a survival benefit, its success is intrinsically linked to meticulous patient selection, precise surgical technique, and rigorous perioperative management in high-volume centers Karimi et al. The insights provided may help optimizing patient safety and standardizing care protocols to minimize postoperative complications.

## Synergistic multimodal treatments

Moving beyond the abdominal cavity, pulmonary oligometastases represent another specific clinical scenario where local therapy can significantly impact survival. Sun et al. presented a retrospective analysis investigating the role of targeted therapy in sensitizing stereotactic body radiotherapy (SBRT). Their study showed that combining concurrent chemoradiotherapy with cetuximab or bevacizumab significantly improved local control, progression-free survival, and overall survival in patients with pulmonary oligometastases from CRC. These findings suggest that the integration of targeted agents can overcome the radioresistance often observed in CRC metastases, providing a rationale for the broader application of combination regimens in the oligometastatic setting Sun et al. This underscores the evolving role of multidisciplinary teams in managing extra-abdominal disease spread through the synergy of systemic and local ablative treatments.

## Predictive modeling and early detection

Finally, the importance of early detection cannot be overstated. While therapeutic options expand, the prognosis of CRC remains inextricably linked to the stage at diagnosis. Yu et al. explored the application of machine learning (ML) to routine blood test indicators for the rapid detection of liver metastasis risk. They achieved high classification accuracy through the implementation of the AdaBoost algorithm, identifying key biomarkers that distinguish CRC patients with and without liver metastasis. This study suggests the potentials of artificial intelligence to transform routine clinical data into powerful predictive tools, offering a non-invasive and cost-effective method for early screening that could significantly improve patient outcomes through timely intervention Yu et al. This approach represents a shift toward precision medicine, utilizing accessible data to stratify patient risk more effectively.

## Conclusion

The articles assembled in this research topic illustrate the dynamic nature of CRC treatment. From the operating room to the radiation suite and the diagnostic laboratory, the integration of novel surgical adjuncts, the refinement of aggressive cytoreductive techniques, the strategic use of targeted sensitizers, and the adoption of advanced computational models collectively advance our ability to combat this disease. Future efforts must continue to focus on personalizing these multimodal approaches to maximize survival while preserving the quality of life for patients.

